# New Diagnosis of B-cell Follicular Lymphoma Temporally Associated With Routine Vaccination: A Report of Two Cases

**DOI:** 10.7759/cureus.106725

**Published:** 2026-04-09

**Authors:** Tina Emami, Debvarsha Mandal, Chidalu Christabel Anyika, Yusuf Salim Khan, Lokesh Pendyala, Muhammad M Salim

**Affiliations:** 1 Research, Avalon University School of Medicine, Willemstad, CUW; 2 Research, Midwestern University Arizona College of Osteopathic Medicine, Glendale, USA; 3 Pulmonology, Chandler Regional Medical Center, Chandler, USA

**Keywords:** covid-19 vaccination, dlbcl, follicular lymphoma, glyphosate exposure, immune activation, lymphadenopathy, lymphoid malignancy, mosunetuzumab, r-chop chemotherapy

## Abstract

Follicular lymphoma (FL) is an indolent B-cell non-Hodgkin lymphoma with potential for transformation into diffuse large B-cell lymphoma (DLBCL). While transient lymphadenopathy is a known response to vaccination, particularly with mRNA COVID-19 vaccines, recent case reports suggest that immune activation may potentially unmask previously undocumented subclinical lymphoproliferative disorders. We report two cases of newly diagnosed FL following routine adult vaccinations. Case 1 involves a 69-year-old male with no prior history of malignancy or immunosuppression, who developed bilateral axillary lymphadenopathy four weeks after receiving the Moderna COVID-19 vaccine, influenza, and pneumococcal vaccines. Excisional biopsy revealed Grade 1-2 FL with a t(14;18)(q32;q21) translocation, and he achieved complete remission following mosunetuzumab therapy. Case 2 describes a 70-year-old male with no prior history of malignancy or immunosuppression, who developed mild, nonspecific symptoms approximately four weeks after completing a two-dose Pfizer COVID-19 vaccine series, with subsequent progression to symptomatic lymphadenopathy. He was diagnosed with FL that had undergone histologic transformation to Stage IV DLBCL and was successfully treated with R-CHOP (rituximab, cyclophosphamide, hydroxydaunorubicin, oncovin, prednisone) protocol and radiation. These reports illustrate the ability of immune stimulation by vaccination to unmask existing but asymptomatic lymphoid malignancies. While vaccination is not implicated as an oncogenic driver, the resulting inflammatory response may serve as a diagnostic “stress test” for the lymphoid system, revealing underlying pathology.

## Introduction

Follicular lymphoma (FL) is a mature B-cell lymphoma that arises from the germinal center B lymphocytes within lymph nodes and forms follicle-like nodules. It is a type of non-Hodgkin lymphoma that usually presents in late adulthood as a painless lymphadenopathy. It is driven by t(14;18)(q32;q21). *BCL2* on chromosome 18 translocates to the Ig heavy chain locus on chromosome 14, resulting in an overexpression of BCL2, which inhibits apoptosis. This blocks the normal apoptotic program of germinal center B cells, allowing survival and accumulation of further genetic alterations [1]. FL occurs in about two to three individuals per 100,000 each year in the United States, ranking as the second most prevalent form of non-Hodgkin lymphoma [2]. Clinically, patients present with painless generalized lymphadenopathy, fatigue, night sweats, fever, and weight loss in more advanced cases.

Large B-cell lymphomas are also a type of non-Hodgkin lymphoma characterized by the proliferation of large neoplastic B lymphocytes. There are different subtypes, with the most common being diffuse large B-cell lymphoma (DLBCL). DLBCL typically presents with rapidly enlarging lymphadenopathy, extranodal involvement, or both, with most patients requiring prompt therapy due to the aggressive clinical course [3]. These lymphomas are characterized by large lymphoid cells expressing pan-B-cell markers (e.g., CD19, CD20). Subclassification is based on techniques such as immunohistochemistry, flow cytometry, and molecular profiling. Gene expression analysis differentiates the germinal center B-cell-like (GCB) subtype from the activated B-cell-like (ABC) subtype, each with distinct genetic profiles and prognostic implications. The ABC subtype is typically linked to a poorer prognosis [3].

FL typically follows a slow-progressing (indolent) course, but, in some patients, the disease can progress early or transform into a more aggressive form, such as DLBCL, which is linked to a worse prognosis [4,5]. FL has an annual transformation rate to DLBCL of 2-3% per year, with cumulative risk increasing over time [6]. The diagnosis of FL is established via excisional biopsy of an involved lymph node, followed by comprehensive evaluation by a hematopathologist. This assessment includes morphological examination, immunohistochemistry, and supplementary studies such as flow cytometry, molecular analysis, and fluorescence in situ hybridization (FISH), which typically reveal a nodular architecture consisting of centrocytes and centroblasts. Excisional biopsy is preferred over core needle or fine-needle aspiration, as the latter often does not provide adequate tissue for thorough pathological assessment or precise classification. Immunophenotyping generally shows expression of B-cell antigens such as CD19 and CD20, along with germinal center markers such as CD10, BCL6, and BCL2. In about 70-95% of cases, the t(14;18) translocation is present, resulting in *BCL2* overexpression; however, some patients may lack this genetic alteration and instead display other molecular changes [1,7].

Both FL and large B-cell lymphoma can also affect extramedullary organs such as the spleen and bone marrow. Although both types of lymphoma are defined by genetic alterations, their development can be influenced by various contributing factors. These include infections, persistent immune activation in immunocompromised individuals (such as those with HIV), age-related cellular changes, spontaneous genetic errors during cell replication, and exposure to certain environmental agents such as radiation or industrial chemicals (e.g., insecticides, Agent Orange). On rare occasions, strong immune responses following vaccination have been speculated as possible contributors. Post-vaccine lymphadenopathy has been observed in roughly 15-65% of individuals following mRNA COVID-19 vaccination, with imaging most frequently detecting it during the initial weeks, particularly among younger individuals and after receiving the second dose. Although the occurrence is less common in older adults and cancer patients, it remains clinically significant.

There have been isolated case reports suggesting a potential link between COVID-19 mRNA vaccination and accelerated progression of angioimmunoblastic T-cell lymphoma (AITL). One such report described a patient with a recent AITL diagnosis who experienced unusually rapid enlargement of lymphomatous lesions following the administration of a BNT162b2 mRNA vaccine booster. This observation has led to the hypothesis that immune activation following vaccination could influence lymphomas derived from T follicular helper cells; however, this remains speculative and is based on limited clinical data [8].

We present two cases of B-cell lymphoma occurring shortly after mRNA COVID-19 vaccination in elderly males: one diagnosed as low-grade FL and the other as transformed DLBCL. This report discusses whether acute immune stimulation could have unmasked subclinical disease.

## Case presentation

Case 1

A 69‑year‑old male with a history of obstructive sleep apnea and no prior diagnosis of cancer, autoimmune disease, or chronic inflammatory condition presented with bilateral axillary lymphadenopathy. He denied tobacco and alcohol use and reported occupational exposure to glyphosate (Roundup), but no other notable environmental exposures. In December 2023, approximately one month before symptom onset, he received three adult vaccinations: the Moderna COVID-19 vaccine in the right deltoid and both influenza and pneumococcal vaccines in the left deltoid.

Clinical Presentation

Four weeks after vaccination, the patient noted painless, persistent swelling in both axillae. He denied any constitutional B symptoms, including fever, night sweats, or weight loss. Physical examination confirmed bilateral axillary lymphadenopathy, with no additional palpable nodes or organomegaly.

Diagnostic Evaluation

Following the development of persistent, painless bilateral axillary swelling, the patient underwent an excisional biopsy of a left axillary lymph node. Histopathologic examination confirmed the diagnosis of FL. Immunohistochemistry demonstrated neoplastic B cells positive for CD19, CD20, CD10, BCL2, and BCL6, consistent with a germinal center B-cell phenotype.

Cytogenetic analysis of the biopsy specimen revealed the t(14;18)(q32;q21) chromosomal translocation, resulting in the *IGH-BCL2* rearrangement, which drives *BCL2* overexpression, a molecular hallmark of FL.

**Figure 1:**
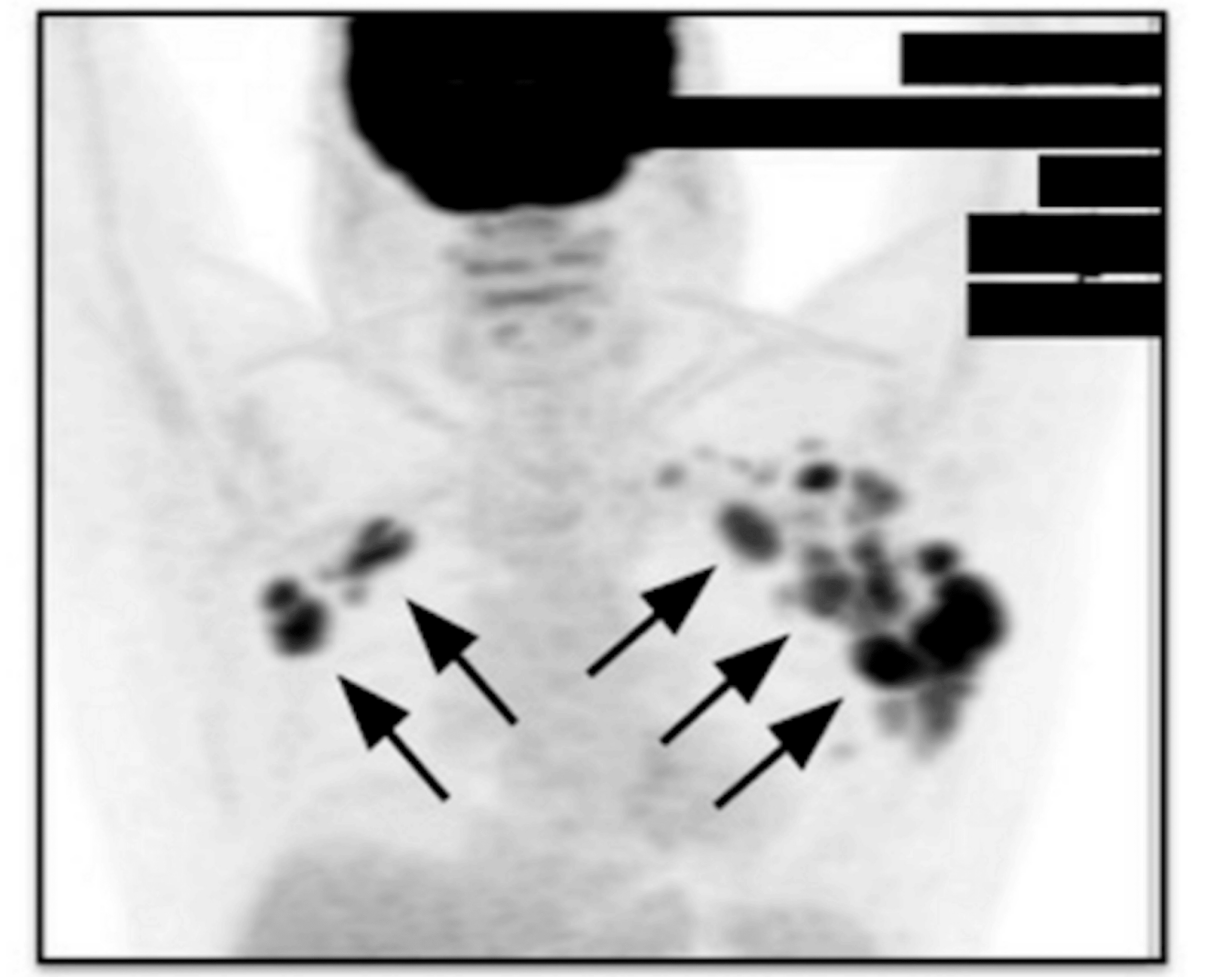
Positron emission tomography (PET) scan before mosunetuzumab treatment (February 15, 2024). PET scan showing bilateral axillary lymphadenopathy with increased fluorodeoxyglucose uptake (maximum standardized uptake value: 11.4), consistent with active lymphoma (arrows).

A positron emission tomography/computed tomography (PET/CT) scan performed on February 15, 2024, was used for staging and showed intensely fluorodeoxyglucose (FDG)-avid lymphadenopathy localized to both axillae, without evidence of disease in other lymph node regions or extranodal sites (Figure 1).

Staging and Pathology

The lymphoma was classified as Stage III due to involvement of lymph node regions on both sides of the diaphragm without extranodal disease or B symptoms.

Histopathologic evaluation showed neoplastic follicles composed mainly of small cleaved centrocytes and occasional centroblasts, with preserved follicular dendritic cell networks, features typical of low-grade follicular lymphoma. The final diagnosis was Grade 1-2 FL, Stage IIIA, germinal center B-cell phenotype.

Before treatment (February 2024), blood counts and chemistry were mostly normal. For example, hemoglobin was 15.6 g/dL, and the white blood cell (WBC) count was 4.4 × 10⁹/L. The patient was clinically stable, but lymphoma was already present on imaging.

During treatment (March 2024), lymphocytes dropped sharply from 1.52 to 0.28 × 10⁹/L, and platelets fell from 104 to 68 × 10⁹/L, consistent with immune stimulation and treatment effect. Liver enzymes rose (aspartate aminotransferase (AST) increased from 33 to 112 U/L, alanine aminotransferase (ALT) increased from 45 to 114 U/L), and lactate dehydrogenase (LDH) increased slightly (220 to 227 U/L), reflecting temporary inflammation and higher cell turnover.

After treatment (June 2025), counts and enzymes normalized with lymphocytes at 1.31 × 10⁹/L, platelets at 107 × 10⁹/L, and AST and ALT back to normal, showing recovery and resolution of the tumor activity (Table 1).

**Table 1:** Summary of laboratory findings before, during, and after treatment (Case 1). No viral testing was performed. ALT: alanine aminotransferase; AST: aspartate aminotransferase; BUN: blood urea nitrogen; eGFR: estimated glomerular filtration rate; LDH: lactate dehydrogenase; MCV: mean corpuscular volume; RDW: red cell distribution width; WBC: white blood cell

Parameter	Pre-treatment (2/2024)	During treatment (3/2024)	Post-treatment (6/2025)	Units	Reference range
Complete blood count					
Hemoglobin	15.6	15.9	15.8	g/dL	13.5–17.5
Hematocrit	46	44.4	46.1	%	38–50
RBC count	5.02	4.87	5.05	×10¹²/L	4.3–5.9
WBC count	4.4	3.8	5.4	×10⁹/L	4.0–11.0
Neutrophils (absolute)	2.26	2.67	3.08	×10⁹/L	1.8–7.0
Lymphocytes (absolute)	1.52	0.28 (low)	1.31	×10⁹/L	1.0–4.0
Platelets	104 (low)	68 (low)	107 (low)	×10⁹/L	150–450
MCV	91.6	91.2	91.3	fL	80–100
RDW	12.7	13.2	13.1	%	11.5–14.5
Comprehensive metabolic panel					
Sodium	142	138	141	mmol/L	135–145
Potassium	4.5	4.4	4.5	mmol/L	3.5–5.0
Chloride	103	101	103	mmol/L	98–107
Bicarbonate (CO₂)	26	27	27	mmol/L	22–29
BUN	11.8	16.6	13.3	mg/dL	7–20
Creatinine	1.05	1.15	1.01	mg/dL	0.6–1.3
eGFR	77	69	81	mL/min/1.73 m²	>60
Calcium	9.9	9.3	9.5	mg/dL	8.5–10.5
Glucose	95	90	98	mg/dL	70–99 (fasting)
Albumin	4.8	4.4	4.8	g/dL	3.5–5.0
Total protein	6.8	6.7	6.9	g/dL	6.0–8.3
Total bilirubin	0.7	1.3 (high)	0.9	mg/dL	0.2–1.2
Alkaline phosphatase	79	127	80	U/L	44–147
AST (SGOT)	33	112 (high)	32	U/L	10–40
ALT (SGPT)	45	114 (high)	44	U/L	7–56
LDH	220	227 (high)	221	U/L	122–222

Treatment and Clinical Course

**Figure 2:**
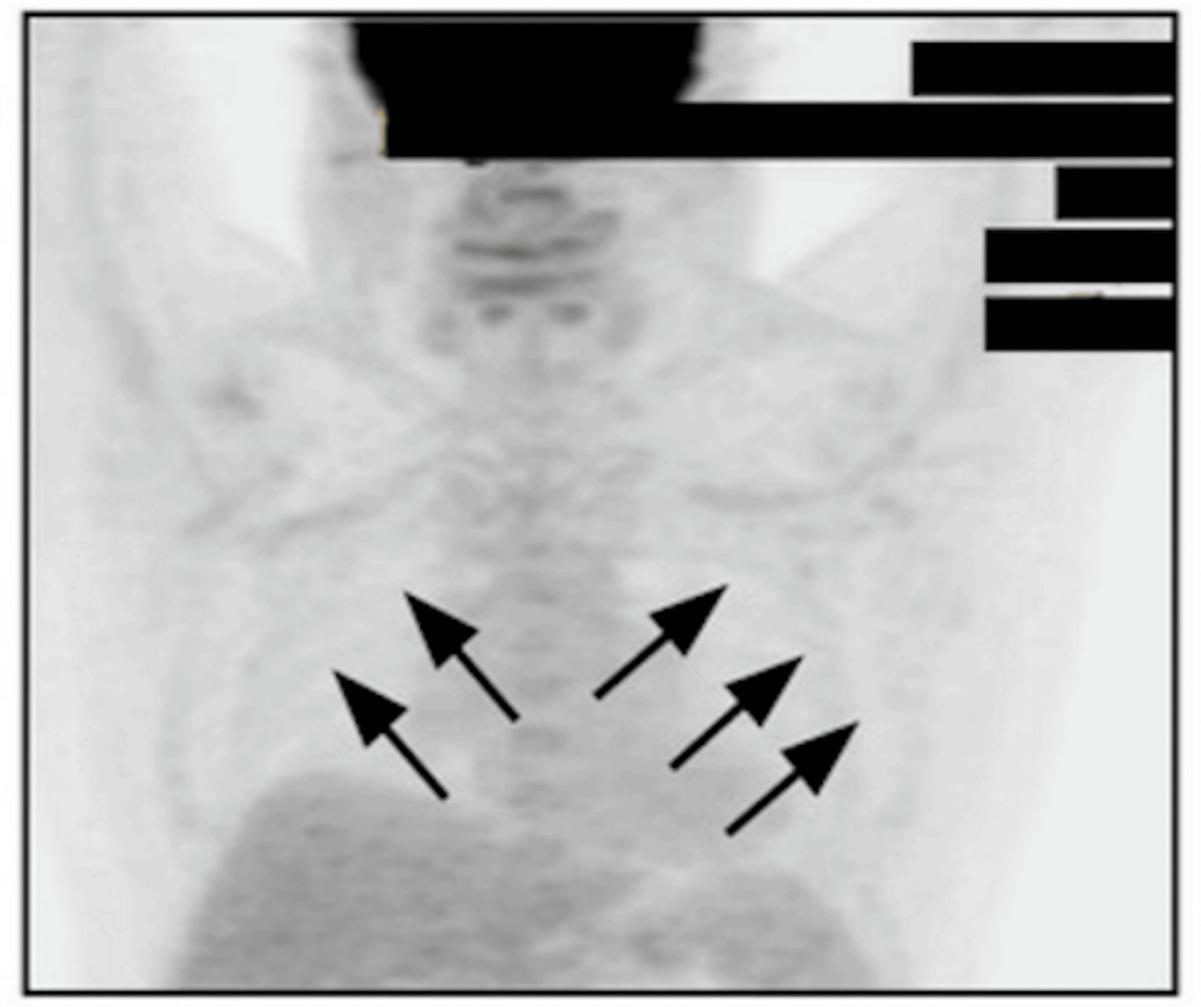
Positron emission tomography (PET) scan after mosunetuzumab treatment (November 25, 2024). Follow-up PET scan showing resolution of fluorodeoxyglucose uptake in the bilateral axillary lymph nodes (maximum standardized uptake value: 1.3), consistent with complete metabolic response (arrows).

The patient was enrolled in the MorningSun clinical trial (NCT05207670) and received 17 cycles of mosunetuzumab, a CD20×CD3 bispecific antibody, as part of a clinical trial protocol rather than standard first-line therapy. He completed treatment in February 2025 without severe adverse events and achieved complete metabolic remission. Serial PET/CT imaging demonstrated complete resolution of previously FDG-avid bilateral axillary lymph nodes by November 2024, consistent with sustained metabolic remission (Figure 2). He has resumed all prior physical activities.

Case 2

A 70-year-old Asian male with a past medical history of prediabetes and hyperlipidemia presented in January 2022 with progressive, painless lymphadenopathy that began initially all over the body. He reported no family history of cancer, autoimmune disease, or hematologic disorders. He had no prior surgical history and denied any known exposure to environmental toxins or allergens. He reported no tobacco, alcohol, or recreational drug use. His childhood vaccinations were fully up to date, and no revaccinations or boosters were administered in the 12 months before diagnosis. The patient denied any recent travel and stated that he seldom traveled overall. The patient’s outpatient medications at the time of presentation included metformin 500 mg, atorvastatin 10 mg, tamsulosin 0.4mg, and low-dose aspirin 81 mg, which he reported taking prophylactically. He demonstrated consistent adherence to his medication regimen and appeared competent in managing his chronic conditions independently. He denied any history of tuberculosis, Epstein-Barr virus, cytomegalovirus, HIV, or any other immunosuppressive diseases or conditions. He had also never received immunosuppressive therapies before his current case diagnosis.

Clinical Presentation

The patient began experiencing mild, nonspecific symptoms approximately one month after completing a two-dose Pfizer-BioNTech COVID-19 mRNA vaccination series in September 2021. Over time, these symptoms gradually evolved to include B symptoms, such as night sweats. Because the early manifestations were mild, he did not seek immediate evaluation. By January 2022, he developed progressive abdominal fullness and distension, accompanied by a palpable upper abdominal mass on physical examination, consistent with bulky intra-abdominal lymphadenopathy. Imaging later confirmed a large mesenteric lymph node conglomerate corresponding to this mass. Additionally, less prominent peripheral lymphadenopathy was observed on examination, including mild involvement of the bilateral axillae and upper extremities; these sites were not prominent on PET/CT imaging, which primarily demonstrated the dominant intra-abdominal disease. The patient remained otherwise clinically stable, without overt signs of systemic illness.

Diagnostic Evaluation

**Figure 3:**
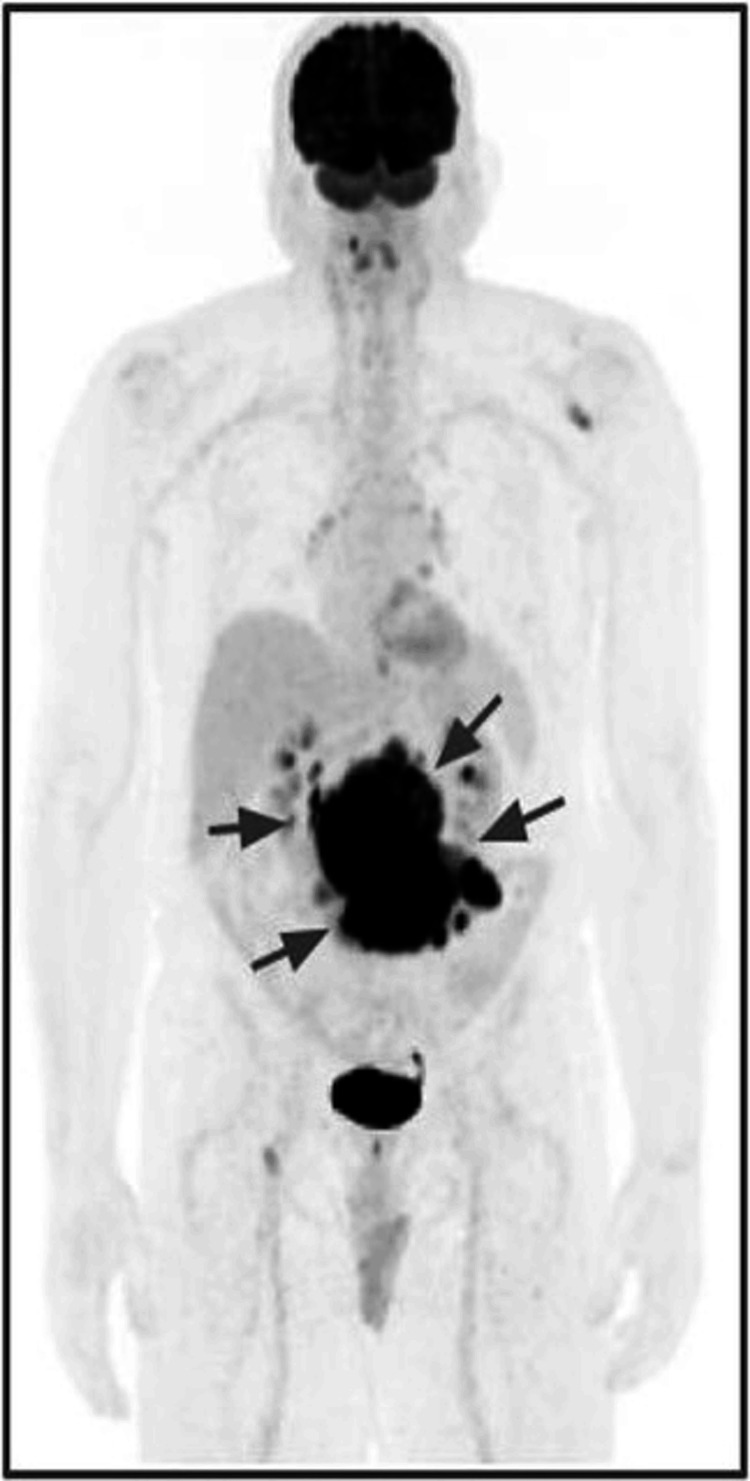
Positron emission tomography (PET) scan before R-CHOP treatment (January 5, 2022). PET scan showing a mesenteric lymph node conglomerate with intense fluorodeoxyglucose uptake (maximum standardized uptake value: 46), consistent with active lymphoma (arrows).

In January 2022, the patient underwent PET/CT imaging (Figure 3) and excisional biopsy of a mesenteric lymph node. The biopsy confirmed follicular B-cell lymphoma with histologic transformation to Stage IV DLBCL.

Immunohistochemistry showed a high-grade B-cell immunophenotype consistent with transformation. Histopathological analysis demonstrated CD20-positive DLBCL of germinal center phenotype, with a Ki-67 proliferative index estimated at 80%-90%. *BCL2* rearrangement was detected in 84% of nuclei, and FISH confirmed the presence of a *BCL2* gene rearrangement. No rearrangement of *MYC* or *BCL6* and no fusion of *MYC* and *IGH* was observed.

Before treatment (January 2022), blood counts and chemistry were mostly normal. Hemoglobin was 14.6 g/dL, WBC was 7.1 × 10⁹/L, and platelets was 241 × 10⁹/L. LDH was mildly elevated at 287 U/L, consistent with active lymphoma. Electrolytes, liver enzymes, and kidney function were within normal limits, and viral screening was negative.

During treatment (February 2022), hemoglobin decreased mildly from 14.6 to 12.1 g/dL, with stable WBCs and platelets, suggesting a mild treatment-related effect. No significant changes were seen in metabolic parameters.

After treatment (January 2025), all values normalized, including LDH (reduced to 158 U/L). Hemoglobin was again 14.6 g/dL, WBC was 6.4 × 10⁹/L, and platelets was 171 × 10⁹/L. These findings indicated full hematologic recovery and sustained complete remission (Table 2).

**Table 2:** Summary of laboratory findings before, during, and after treatment (Case 2). —: Infectious/viral screening results are qualitative and do not have units. ALT: alanine aminotransferase; AST: aspartate aminotransferase; BUN: blood urea nitrogen; eGFR: estimated glomerular filtration rate; LDH: lactate dehydrogenase; MCV: mean corpuscular volume; RDW: red cell distribution width; WBC: white blood cell; WNL: within normal limits

Parameter	Pre-treatment (1/2022)	During treatment (2/2022)	Post-treatment (1/2025)	Units	Reference range
Complete blood count					
Hemoglobin	14.6	12.1 (low)	14.6	g/dL	13.5–17.5
Hematocrit	45.2	43	44.8	%	38–50
RBC count	4.92	4.7	4.87	×10¹²/L	4.3–5.9
WBC count	7.1	WNL	6.4	×10⁹/L	4.0–11.0
Neutrophils (absolute)	WNL	WNL	3.69	×10⁹/L	1.8–7.0
Lymphocytes (absolute)	WNL	WNL	1.9	×10⁹/L	1.0–4.0
Platelets	241	WNL	171	×10⁹/L	150–450
MCV	91.9	90	92	fL	80–100
RDW	12.6	12.6	12.1	%	11.5–14.5
Comprehensive metabolic panel					
Sodium	139	WNL	137	mmol/L	135–145
Potassium	4.1	WNL	4.4	mmol/L	3.5–5.0
Chloride	99	WNL	101	mmol/L	98–107
Bicarbonate (CO₂)	28	WNL	26	mmol/L	22–29
BUN	12.9	WNL	14.5	mg/dL	7–20
Creatinine	0.91	WNL	1.03	mg/dL	0.6–1.3
eGFR	88	WNL	79	mL/min/1.73 m²	>60
Calcium	9.4	WNL	8.8	mg/dL	8.5–10.5
Glucose	77	WNL	128 (high)	mg/dL	70–99 (fasting)
Albumin	4.7	WNL	4.3	g/dL	3.5–5.0
Total protein	7.1	WNL	6.4	g/dL	6.0–8.3
Total bilirubin	0.2	WNL	0.4	mg/dL	0.2–1.2
Alkaline Phosphatase	77	WNL	74	U/L	44–147
AST (SGOT)	19	WNL	20	U/L	10–40
ALT (SGPT)	12	WNL	20	U/L	7–56
LDH	287 (high)	WNL	158	U/L	122–222
Infectious / Viral Screening					
HIV-1/2 antigen/antibody	Negative	Negative	Negative	—	Negative
Hepatitis B surface antigen	Negative	Negative	Negative	—	Negative
Hepatitis B core antibody	Negative	Negative	Negative	—	Negative
Hepatitis B surface antibody	Negative	Negative	Negative	—	Positive/Immune
Hepatitis C antibody	Negative	Negative	Negative	—	Negative
SARS-CoV-2 RNA PCR	Undetected	Undetected	Undetected	—	Undetected

Staging and Pathology

The disease was classified as Stage IV based on disseminated lymphadenopathy and systemic dissemination.

Treatment and Clinical Course

In February 2022, the patient received three cycles of outpatient R-CHOP chemotherapy in combination with targeted radiation therapy. R-CHOP consists of rituximab, a monoclonal anti-CD20 antibody; cyclophosphamide, an alkylating agent; doxorubicin, an anthracycline; vincristine, a microtubule inhibitor; and prednisone, a corticosteroid.

**Figure 4:**
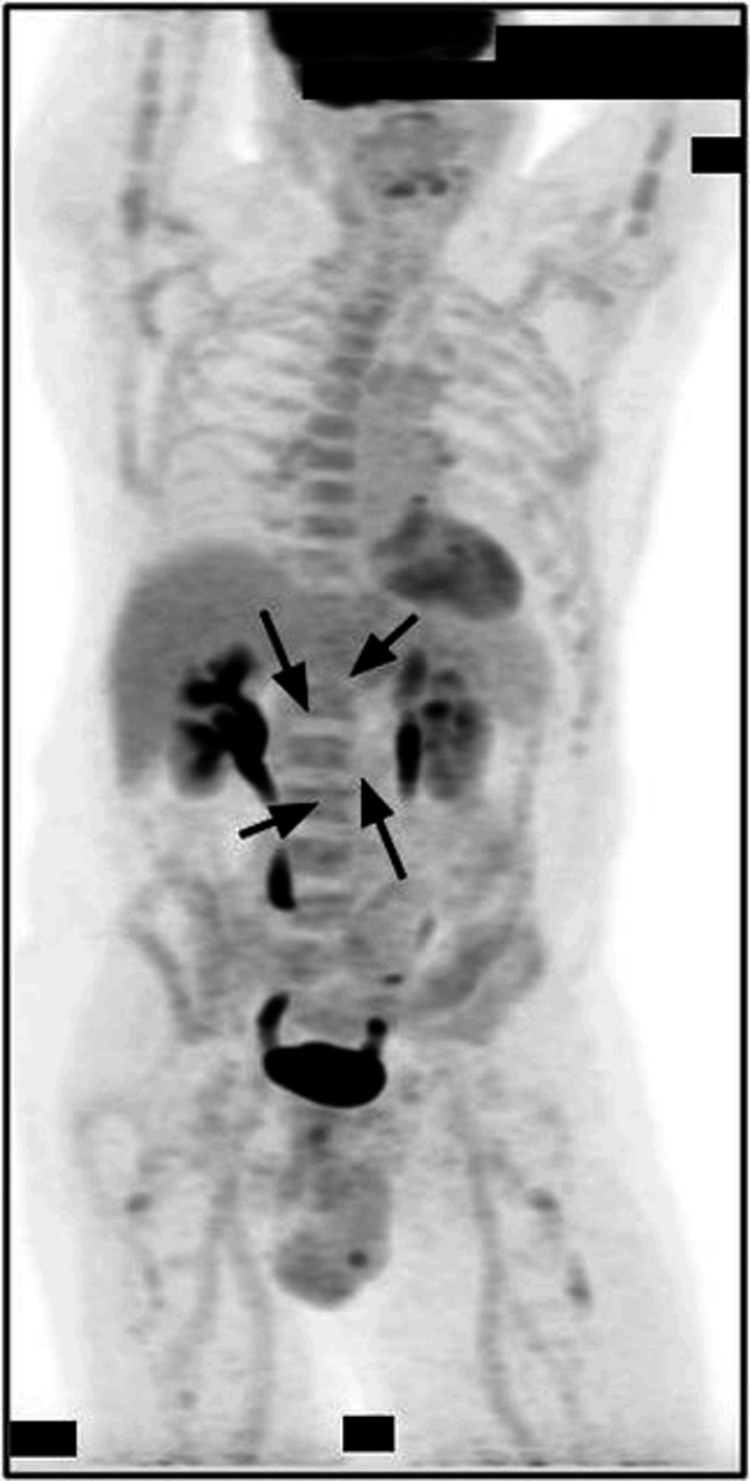
Positron emission tomography (PET) scan after R-CHOP treatment (May 26, 2022). Follow-up PET scan showing resolution of fluorodeoxyglucose uptake in the mesenteric lymph node conglomerate (maximum standardized uptake value: 2.6), consistent with treatment response (arrows).

The patient experienced self-limited minimal fatigue during chemotherapy and did not require medical intervention. He did not present with any relevant adverse effects, such as cytopenias requiring transfusion, infections, or neuropathy. Post-therapy PET/CT imaging three months after treatment showed resolution of FDG uptake of the mesenteric lymph node conglomerate (Figure 4). No pulmonary masses, abdominal organ involvement, or acute osseous abnormalities were noted. Overall, the findings were consistent with no definitive evidence of active lymphoma. ​​Follow-up CT abdomen and pelvis with intravenous contrast showed no evidence of abdominopelvic or inguinal lymphadenopathy, and all major abdominal organs, including the liver, pancreas, spleen, kidneys, adrenals, and gallbladder, appeared normal, aside from stable, benign-appearing liver and renal cysts.

He continues to be in complete clinical remission, with normalization of energy status and absence of recurrence of lymphadenopathy. He has resumed all prior physical activities.

## Discussion

This is a case series of two older male patients diagnosed with B-cell lymphomas, one of low-grade FL and the other of histologically transformed DLBCL, shortly after mRNA COVID-19 vaccination. The patients differed by vaccine exposure (Moderna versus Pfizer) and aggressiveness at disease presentation. Both patients developed symptoms approximately one month after vaccination. One patient also had long-term occupational glyphosate-containing herbicide exposure, a potential environmental risk factor for B-cell lymphomas. The described cases raise clinically relevant questions regarding whether transient immune activation following vaccination could reveal pre-existing, asymptomatic lymphoid malignancies in patients with other risk factors. While post-vaccination lymphadenopathy is a well-recognized benign immune response, persistent or diffuse lymphadenopathy, especially after four to six weeks, needs to be carefully evaluated to exclude malignancy. While temporal proximity does not establish causation, these cases underscore the importance of careful clinical evaluation and reporting when lymphoma is diagnosed following vaccination.

FL is an indolent subtype of non-Hodgkin B-cell lymphoma that originates from germinal center B cells. According to the 2022 World Health Organization (WHO) Classification of Lymphoid Neoplasms, diagnosis is based on immunohistochemistry and cytogenetic profiling [9]. FL is characterized by the t(14;18)(q32;q21) translocation, which juxtaposes the *BCL2* gene to the Ig heavy chain locus, causing the overexpression of *BCL2* and resistance to apoptosis [10]. Additional mutations in genes such as *EZH2*, *CREBBP*, *KMT2D*, and *TNFRSF14* can cause immune evasion and disease progression [5].

Even though FL usually follows an indolent course, histologic transformation to aggressive subtypes such as DLBCL occurs in approximately 15-30% of cases, and it is associated with significantly worse outcomes [11]. Transformation is usually associated with increased cellular proliferation, loss of germinal center markers, and additional genetic mutations such as *MYC* and *TP53* [12].

In both cases, persistent lymphadenopathy developed shortly after COVID-19 mRNA vaccination. In Case 2, the transformation to DLBCL represents a biologically aggressive progression that significantly worsens prognosis and emphasizes the importance of early histological classification to guide timely initiation of appropriate therapy.

Post-vaccination lymphadenopathy, particularly ipsilateral axillary swelling, is a well-documented, benign immune response to mRNA COVID-19 vaccines, typically resolving within four to six weeks [13]. Persistent lymphadenopathy beyond this timeframe should prompt further evaluation to exclude malignancy. Large epidemiologic studies, such as the population-based case-control study by Morton et al. in 2009, have not shown an increased risk of FL or other non-Hodgkin lymphomas associated with adult vaccination history [14].

However, vaccination-induced immune activation may unmask previously undiagnosed lymphoid malignancies. COVID-19 mRNA vaccines strongly stimulate germinal centers via T-follicular helper (Tfh) cells, inducing B-cell proliferation through interleukin (IL)-4 and IL-21 cytokines [15]. Menter et al. in 2023 showed that such vaccine-induced lymphadenopathy typically involves polyclonal extrafollicular B-cell proliferation [16]. In rare cases, this immune stimulation can potentially accelerate the growth of pre-existing malignant clones, bringing otherwise indolent disease to clinical attention.

Continued antigenic stimulation may also promote T-cell dysfunction and immune exhaustion, which can lead to impaired cytotoxic responses and diminished immune surveillance. Immune dysfunction and an immunosuppressive tumor microenvironment are increasingly recognized as key contributors to B-cell lymphoma development and progression, which supports malignant B-cell survival and clonal expansion [17]. Persistent immune activation and a dysregulated tumor-host immune interaction may therefore contribute to lymphomagenesis in susceptible individuals.

Some case reports highlight such instances of lymphomas being diagnosed shortly after COVID-19 vaccination. Tintle et al. in 2021 described florid lymphoid and Langerhans cell hyperplasia mimicking lymphoma [18]. Patil et al. in 2022 reported a case of atypical follicular hyperplasia with light chain restriction, raising concern for FL [19]. Additionally, Goldman et al. in 2021 documented rapid progression of AITL following mRNA vaccination [8,20]. While these observations remain anecdotal, they bring to light the need for vigilance when lymphadenopathy persists post-immunization.

Environmental exposure may have contributed to lymphomagenesis in Case 1. Pooled agricultural cohort studies and meta-analysis have shown that glyphosate-based herbicides have been associated with an increased risk of non-Hodgkin lymphoma [9,10]. While causality can remain debated, prolonged occupational exposure represents a recognized epidemiologic risk factor. Hence, in this context, the patient’s long-term glyphosate exposure may have constituted a predisposing factor, with immune activation that may have facilitated clinical detection, acting synergistically with vaccine-related immune activation, rather than serving as a primary etiologic driver.

Both patients expressed concern and anxiety regarding the temporal association between their vaccinations and lymphoma diagnosis. Therefore, it is important to address patients’ concerns through clear, evidence-based communication delivered with empathy and transparency to avoid unnecessary vaccine hesitancy and to reinforce the established safety profile of the vaccination.

Fortunately, both patients received treatments that are of a high clinical standard and achieved remission. The first patient was treated with mosunetuzumab, a bispecific CD20×CD3 antibody that directs T-cell-mediated cytotoxicity against malignant B cells [20]. The second patient was treated with R-CHOP chemotherapy and radiation, the current first-line regimen for DLBCL [21].

Surveillance recommendations

Follow-up post-treatment should follow standard guidelines, but possibly requires individualized timing based on clinical context. For FL, the existing National Comprehensive Cancer Network guidelines recommend clinical evaluation every three to six months for five years, with imaging reserved for symptom-directed assessment and not routine follow-up [22]. For DLBCL, formal imaging (e.g., PET/CT) every three to six months for the first two years with annual review thereafter is advised [23]. Based on the unusual detection of the disease shortly after vaccination, physicians can consider altering the follow-up duration based on patient anxiety and disease aggressiveness.

Limitations and clinical implications

The observational nature, small sample size, and lack of pre-vaccination baseline imaging or laboratory data are the biggest limitations of this case report. These cases emphasize the clinical importance of evaluating persistent lymphadenopathy after vaccination, especially in elderly patients or those with known environmental exposures. Immune stimulation may unmask latent lymphoid malignancies, but current evidence does not support a causal relationship and reinforces that vaccination remains overwhelmingly safe and essential. Hence, clinicians should balance appropriate evaluation with careful communication to avoid promoting unnecessary vaccine hesitancy.

## Conclusions

We describe two cases of B-cell lymphoma diagnosed shortly after vaccination with the COVID-19 mRNA vaccine, one involving low-grade FL and the second a histologically transformed DLBCL. Although no causality can be determined, these cases highlight that previously unconfirmed lymphoid malignancies may become clinically apparent in certain individuals, especially in older patients or those with environmental exposures. Prolonged or widespread lymphadenopathy after vaccination should raise the suspicion for prompt clinical workup, including histopathologic and imaging evaluations, to facilitate early diagnosis and appropriate intervention. Recognition of this uncommon but clinically relevant situation can inform clinicians on how to strike the appropriate balance between vigilant monitoring and patient reassurance. Notably, these reports should not deter vaccination, which is safe and critical to public health.
